# Cross-sectoral integration in youth-focused health and social services in Canada: a social network analysis

**DOI:** 10.1186/s12913-018-3742-1

**Published:** 2018-11-28

**Authors:** Rachel McGihon, Lisa D. Hawke, Gloria Chaim, Joanna Henderson

**Affiliations:** 1 Centre for Addiction and Mental Health, 80 Workman Way, Toronto, Ontario M6J 1H4 Canada; 2 Department of Psychiatry, University of Toronto, Toronto, Ontario Canada; 3 Dalla Lana School of Public Health, University of Toronto, Toronto, Ontario Canada

**Keywords:** Youth, Mental health, Substance use, Concurrent disorder, Service integration

## Abstract

**Background:**

Youth with concurrent substance use and mental health concerns have diverse psychosocial needs and may present to a multitude of clinical and social service sectors. By integrating service sectors at a system level, the diversity of needs of youth with concurrent disorders can be addressed in a more holistic way. The objective of the present study was to quantify the level of cross-sectoral integration in youth-focused services in Canada.

**Methods:**

Social network analysis (SNA) was used to examine the relationships between eight sectors: addictions, child welfare, education, physical health, housing, mental health, youth justice, and other social services. A total of 597 participants representing twelve networks of youth-serving agencies across Canada provided information on their cross-sectoral contacts and referrals.

**Results:**

Overall, results suggested a moderate level of integration between sectors. The mental health and the addictions sectors demonstrated only moderate integration, while the addictions sector was strongly connected with the youth justice sector.

**Conclusions:**

Despite evidence of moderate integration, increased integration is called for to better meet the needs of youth with concurrent mental health and substance use concerns across youth-serving sectors. Ongoing efforts to enhance the integration between youth-serving sectors should be a primary focus in organizing networks serving youth with concurrent mental health and substance use needs.

## Background

Mental health concerns affect a large proportion of youth and young adults. An estimated 11% of Canadians aged 15 to 24 have experienced depression in their lifetime [1] and some 12.6% of children and youth are estimated to have a clinical mental health or substance use disorder at any time [2]. Mental disorders frequently co-occur with substance use problems in young persons [3]. In youth service settings specifically, some 41% of service-seeking youth experience comorbidity [4]. Co-occurring mental health and substance use disorders, or concurrent disorders (CDs), in early life may exacerbate negative outcomes, which include impaired social and psychological functioning [5], increased risk of academic problems and suicidality [6, 7]. The potential long-term effects of CDs in youth underscore the need for early intervention through developmentally-appropriate and evidence-based practices [8, 9]. Our previous work illustrates that many youth accessing youth-serving agencies across sectors have CDs and multiple intersecting needs [4, 10, 11]. Unfortunately, many individuals with CDs report a perceived unmet need for care [12] despite frequent interactions with mental health and addictions services [13].

This unmet need has been attributed, in part, to flaws in existing youth mental health systems. Youth with CDs may present to specialty mental health and addictions treatment centers, or the child welfare, youth justice, education and primary care service sectors [4, 14–16]. Yet, services across these sectors are insufficiently integrated to respond to the diverse and ever-changing needs of youth [17–19]. Fragmentation across sectors may result in significant treatment delays, inconsistent service use, and discontinuity in the provision of care [18, 20]. Addressing these concerns requires achieving system-level integration by strengthening connections between mental health, addictions, health and social services organizations [21, 22].

Eliminating fragmentation through the organization of integrated service networks is expected to improve the responsiveness and efficiency of youth mental health systems [22]. Highly integrated systems are characterized by shared goals, coherent treatment philosophies, and frequent communication and interaction [23, 24]. Youth seeking mental health and addictions services through integrated systems can move seamlessly through individualized care pathways, unrestricted by differences in organizational priorities, funding structures and referral practices [24, 25]. Previous research suggests that inter-agency coordination enhances the ease and timeliness of youth service access [20, 25, 26]. Preliminary evidence also points to the potential for integrated service systems to improve psychosocial functioning. For example, Bai and colleagues (2009) found that greater intensity of inter-organizational relationships – defined by the number of linkages between organizations – predicted an increased likelihood of service use and mental health improvement in children [20].

Quantifying the degree of inter-agency integration in youth mental health systems will help to establish an empirical justification for systems-level reform. The objective of the current study is to quantify the level of cross-sectoral integration in youth-focused service networks using social network analysis (SNA).

## Methods

SNA [27, 28] methods were used to examine the connections between service sectors participating in the National Youth Screening Project (NYSP) [29, 30]. NYSP was funded under Health Canada’s Drug Treatment Funding Program (DTFP) and received ethics approval from the Centre for Addiction and Mental Health in Toronto, Canada, as well as organization-specific review boards for all participating agencies. Informed consent was obtained from all individual participants included in the study.

### Network Bounding & Identification

Service sectors were the actors of interest and the boundary of the network was defined by NYSP participation. That is, the network under consideration was comprised of the sectors that were represented by the service agencies participating in the project; from hereon in, this is referred to as the ‘NYSP networks.’ While the entry point to project invitation was via the addictions sector, invitations were then disseminated to a broad range of service organizations across sectors as part of a CD capacity-building project. Sites interested in participating in NYSP were required to identify service agencies from a minimum of two of nine sectors: addictions, child welfare, education, family services, health services, housing outreach and support, youth justice, mental health, and social services. The final composition of the networks in the study depended on self-selection based on interest and capacity to commit to the time required for the CD-focused project. Full details on the project processes have been published elsewhere [30].

### Data Collection & Measurement

Following recruitment, service providers from participating agencies completed a one-day capacity building session. The focus of these sessions has been described previously [29, 30]. Prior to beginning their session, service providers completed the Service Provider Survey, a self-report questionnaire that was used to collect individual-level demographics (e.g., age, sex, educational background) and measures of inter-agency integration. Networking data were drawn from four questions included in the Service Provider Survey. Service providers were asked to indicate how often during the previous 3 months they had 1) contacted, 2) been contacted by (“contact network”), 3) made referrals to, and 4) received referrals from (“referral network”) each of the youth-serving sectors represented in the NYSP networks. Each type of relation was measured on a 7-point Likert scale ranging from *not at all* (1) to *often* (7). Following the approach that is recommended for combining multiple views when data are valued [31], we grouped all service providers by the self-reported service sector of their agency and then calculated a median score for each sector. A valued variable capturing tie strength was then created for each of the four relations; median scores from 1 to 2 were categorized as *low* (1), scores from 3 to 5 as *moderate* (2) and scores from 6 to 7 as *high* (3).

### Analysis

For the purposes of the SNA, the twelve networks of service providing agencies engaged in NYSP were analyzed as a single network to estimate the overall level of integration of youth-serving agencies across Canada. This is consistent with a *socio-centric* or *whole network approach* that involves the study of all the relational ties among actors of a predefined group [32]. This approach is appropriate when network membership is known a priori [33] and when network boundaries are determined by the methodologies used to identify the network members [34], as was the case for the current study. We generated two data matrices, which were weighted networks with plausible cell values from 2 to 6 to summarize both the supply and demand of information. Visualization of each network was performed in UCINET v6.627 [35] and measures of network structure were calculated using the R package tnet v3.0.14 [36].

#### Service integration

Service integration was measured by *network density*, defined as the average strength of ties within a network and is calculated by taking the sum of the values of all ties and dividing by the number of possible ties [27, 31]. The density for both contact and referral relations was used to quantify the overall level of integration in NYSP [28], where strong average tie strength would indicate a high frequency of interaction between all represented service sectors.

## Results

### Sample characteristics

Sample characteristics are presented in Table 1. Service sector and networking data were available for service providers (*n* = 597) from twelve networks of youth-serving agencies across Canada, including representation from the provinces of British Columbia, Manitoba, Newfoundland, Nova Scotia, Ontario and Prince Edward Island. Service providers represented agencies from addictions (22%), child welfare (8%), education (9%), housing (5%), mental health (25%), physical health (1%) and youth justice (14%) sectors. The remaining 16% were employed by other agencies within the family and/or social service sectors.

**Table 1:** Demographic characteristics of service providers (*n* = 597)

	Number	Percent
Service sector		
Addictions	132	22.1
Child welfare	47	7.9
Education	55	9.2
Housing	32	5.4
Mental health	147	24.6
Physical health	4	0.7
Youth justice	84	14.1
Other	96	16.1
Sex		
Male	115	19.2
Female	467	78.3
Missing	14	2.3
Age		
20–29	82	13.7
30–39	201	33.7
40–39	171	28.6
50–59	116	19.4
60–69	20	3.4
Missing	7	1.2
Highest level of education		
High school diploma	17	2.8
College diploma	81	13.6
Bachelor’s degree	284	47.6
Master’s degree	189	31.7
PhD	7	1.2
Other	13	2.2
Missing	6	1.0

### Service integration

**Fig. 1:**
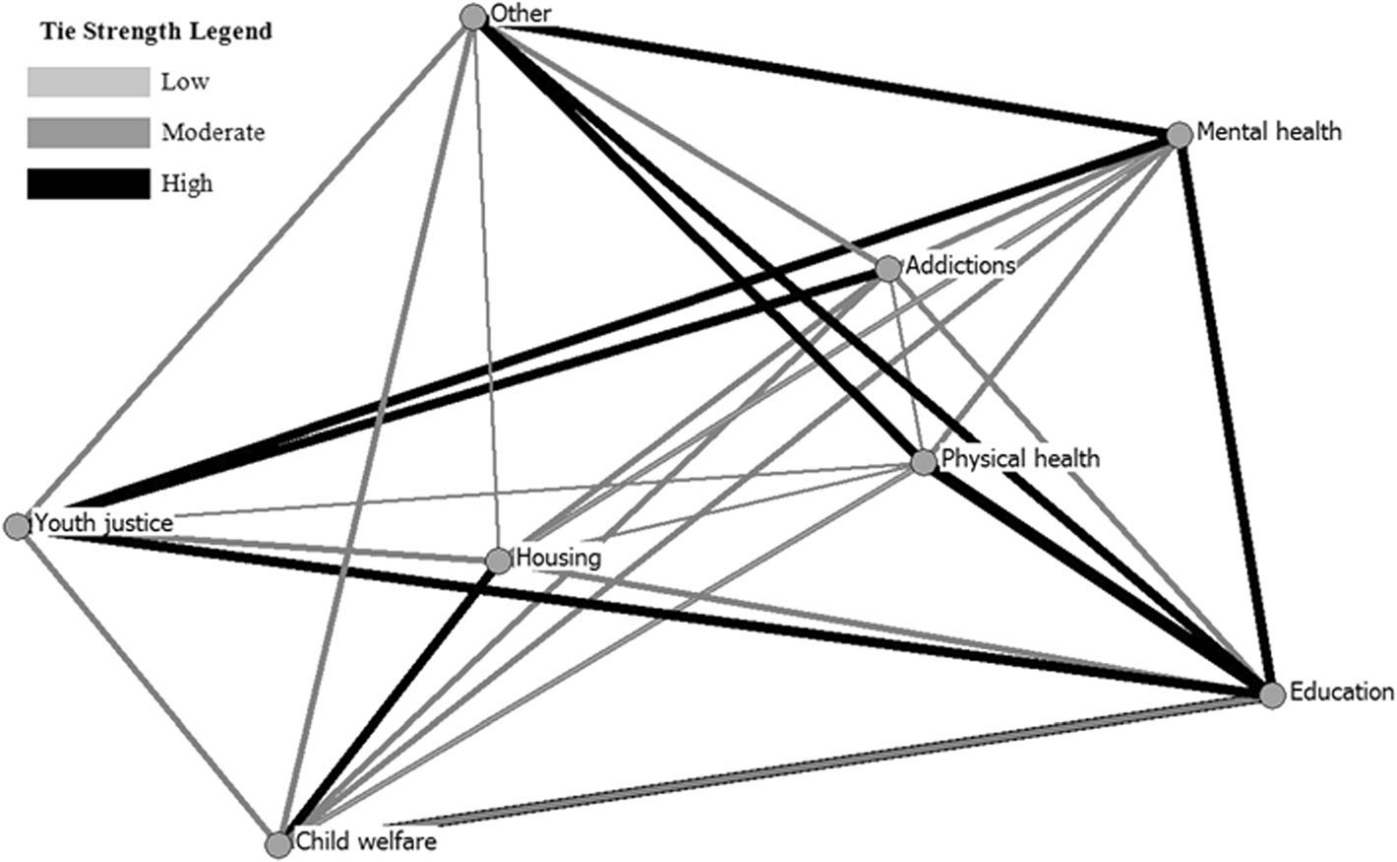
Contact ties in the NYSP networks

The structures of the contact and referral networks are displayed in Fig. 1. Service sectors are represented by circles (nodes) and relational ties are presented in gray scale according to median tie strength (i.e., low, moderate or high). Overall, measures of global density indicated a moderate level of cross-sectoral integration in NYSP; average self-reported frequency of contacts made, contacts received, referrals made and referrals received was in the mid-range. Density in the contact network (3.59 ± 1.07) appeared to be slightly higher than that observed in the referral network (2.77 ± 0.68), which suggests that contact between agencies from different service sectors may have been more common than referrals; however, this difference was not statistically significant (*t*(7) = 1.83, *p* = 0.09).

## Discussion

The present study examined the level of cross-sectoral integration across youth-focused service agencies in Canada. Our parallel work from the same overarching NYSP project shows that CDs and multiple overlapping needs are extremely common among youth seeking services in the sampled organizations [4, 10, 11], highlighting the importance of strong system integration.

**Fig. 2:**
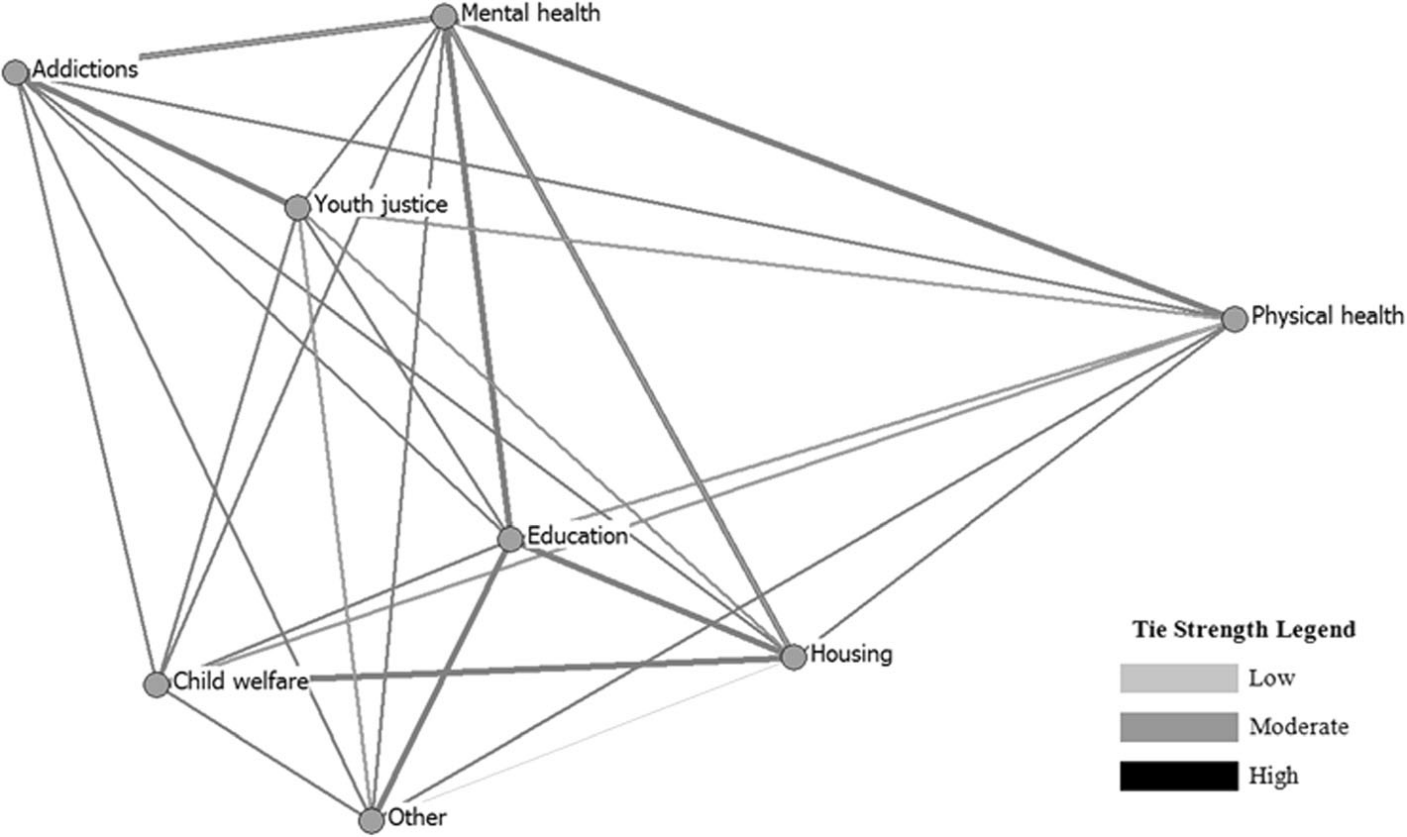
Referral ties in the NYSP networks

Results show that overall, network density was in the mid-range and suggested a fair level of integration between eight different health and social service sectors. We observed only a moderate level of collaboration between the mental health and the addictions sectors (Figs. 1 & 2). This is not to say that these sectors did not engage in collaborative practices; however, given the documented association between substance use and mental health concerns [3, 4, 10], we might expect to see stronger relationships between these two sets of service agencies. These results might reflect service providers’ lack of recognition of the co-occurring needs of their clients, as well as the longstanding tradition of treating substance use and mental disorders in separate service settings [5, 37]. The addictions sector was also strongly connected with youth justice, which may reflect the high level of substance use concerns among youth in the justice system [16], or perhaps a lower threshold in the justice system for connecting with substance use services [38]. In either case, enhancing the linkages between the addictions and mental health sectors should be a primary focus in organizing networks serving youth with CDs and may be an important area for future research and intervention.

These findings are generally consistent with previous work examining patterns of service utilization in service-seeking youth. Among children and adolescents with CDs who are receiving treatment, the majority access services through the school system, followed by the mental health system [39–41]. Relatively fewer access services through other social service sectors [39, 40], although these sectors may receive referrals from mental health practitioners whose clients have overlapping psychosocial needs [42]. The liaison role of the child welfare sector is compatible with its mandate to coordinate the services that are necessary to safeguard the wellbeing of children [43].

A broad body of literature has described the potential client- and organizational-level benefits of well networked, collaborative care systems that focus simultaneously on the many mental, physical and social needs of youth presenting to CD services [44]. In Canada, support for enhanced integration in the delivery of mental health and addictions services is evident in the reflections of clinical, academic and policy professionals [23, 45]. Although this may be achieved at various levels of care – for example, through integrated collaborative care teams or “service hubs” [10, 45] – there is also a need for enhanced integration at the broadest level.

The moderate level of cross-sectoral integration in Canadian youth-serving agencies justifies the need to address residual fragmentation. A systems-level approach emphasizes connections between organizations operating in different service sectors, and is often endorsed as a “gold standard” integrative strategy to supplement those at the level of individual clients and services [46, 47]. Despite its promise, the existing literature does not point to a single model for successful integration, nor is there strong empirical support for specific integration strategies or processes [44, 47]. A number of recommendations guiding the development of integration strategies have emerged from the general health systems literature: the need for standardized referral procedures, indicator-based performance management, effective information systems, and shared organizational culture and leadership [22, 47]. These recommendations are admittedly broad and underscore the need for ongoing research and evaluation to establish an evidence base at this level [44].

The level of integration across multi-sector systems does not necessarily speak to the experiences of youth with CDs. Although integration might be expected to have benefits for clients, considering previous work documenting the efficiency of integrated vs. non-integrated care systems [44], an association between enhanced systems-level integration and improved health outcomes cannot be assumed without supporting empirical evidence. This pathway is likely complicated by many mediating and moderating variables, which highlights the need for additional quantitative and qualitative research to demonstrate the value of integration for youth’s service experiences and health-related outcomes [44, 48]. Evaluations of integrated services and interventions should consider youth and family perspectives, and should include client-level, as well as organizational- and service-specific outcome measures.

This study is strengthened by the use of a whole network approach to investigate the relationships between diverse youth-serving sectors at a national level, thus providing a comprehensive, systems-level picture of cross-sectoral integration. Analyses employed SNA, a distinctive set of methods that allow for the empirical study of structural relations [32, 33]. Whereas relationships between actors in a network are commonly quantified using binary data – as either present or absent, according to a defined threshold – we conducted a weighted network analysis to capture the strength of the connections between pairs of service sectors.

### Limitations

Our findings should also be considered in light of limitations. Notably, this was not a conventional SNA in which participants indicated their degree of connection with other individuals, but rather with other sectors. This may constitute a limitation as a departure from typical methodology; alternatively, it may provide information about inter-sector connectedness that extends beyond individual, personal connections. Second, the Service Provider Survey did not ask respondents to report which specific agency they were working for. As such, we were not able to account for whether agencies were over- or under-represented. In addition, data were from a convenience sample of service providers who expressed interest in and commitment to participating in the NYSP CD-focused project, and results may therefore be subject to selection bias. This may have resulted in an over-representation of individuals who had favorable views of service integration, or who had previously established relationships with service providers or agencies from other sectors. Indeed, certain sectors (e.g., physical health) are under-represented; as efforts to bridge physical health care providers with other sectors advances through progressive initiatives such as Family Health Teams incorporating primary care providers and disciplines such as social work [49], future research should examine the actual impact of these initiatives on cross-sectoral integration.

## Conclusions

Youth with concurrent substance use and mental health concerns have diverse psychosocial needs and may present to a multitude of clinical and social service sectors, justifying the need for cross-sectoral relationships. Across Canadian youth-serving agencies, service sectors appear to be only moderately well connected and there is a need for ongoing efforts to enhance inter-agency integration. Interventions aimed at increasing the level of integration at the systems-level of care should adhere to a broad set of recommendations, but must also take into consideration additional contextual factors shaping integration goals and processes. Overall, integration efforts should adopt a client-focused perspective, focused on improving the coordination and delivery of services for youth with CDs to optimize care for this vulnerable population.

## Abbreviations

CDs: Concurrent Disorders
DTFP: Health Canada’s Drug Treatment Funding Program
SNA: Social Network Analysis
